# Patient satisfaction and discharge-planning quality following upper gastrointestinal endoscopy: a single-center cross-sectional study

**DOI:** 10.3389/fmed.2026.1903766

**Published:** 2026-10-29

**Authors:** Samia Eaid Elgazzar, Mohamed Goda Elbqry, Shereen Ahmed A. Qalawa, Fatima Sayed Ashmieg, Saddam Ahmed Al-Ahdal, Meead Abdulaziz Alsohibani, Fatma Mohamed Elmansy

**Affiliations:** 1 Department of Medical-Surgical Nursing, College of Nursing, Qassim University, Buraydah, Saudi Arabia; 2 Department of Nursing, Medical City, Qassim University, Buraydah, Saudi Arabia; 3 Department of Medical-Surgical Nursing, Faculty of Nursing, Port-Said University, Port-Said, Egypt; 4 Department of Medical-Surgical Nursing, Faculty of Nursing, Suez Canal University, Ismailia City, Egypt; 5 Department of Nursing and Midwifery, Faculty of Medicine and Health Sciences, Al-Razi University, Sana'a, Yemen

**Keywords:** discharge planning, endoscopy, gastrointestinal, patient discharge, patient reported outcome measures, patient satisfaction, quality indicators, quality of health care

## Abstract

**Background:**

Effective discharge planning and patient satisfaction are essential elements of high-quality care after upper gastrointestinal (GI) endoscopy. Adherence to post-procedure protocols and patient outcomes may be enhanced by clear communication and thorough instructions.

**Aim:**

: This study aims to assess patient satisfaction and discharge-planning quality following upper gastrointestinal endoscopy and to examine the association between them.

**Methods:**

A single-center cross-sectional study was conducted at the Digestive Endoscopy Service of Qassim University Medical City, using a convenience sample of 74 patients who underwent upper gastrointestinal endoscopy. Eligible patients were approached in person in the endoscopy unit after the procedure and were provided with a link or QR code to complete a self-administered electronic Arabic questionnaire. We assessed patient satisfaction using the modified Group Health Association of America-9 (mGHAA-9) instrument, and discharge-planning quality using a newly developed discharge-planning questionnaire.

**Results:**

The median primary 8-item discharge-planning score was 7.0 (IQR, 5.0–8.0), corresponding to a median percentage score of 77.8% (IQR, 55.6–88.9%), and the median patient satisfaction score was 33.0 (IQR, 20.0–35.0) among the 74 participants. While 63.5% of individuals received written instructions, 83.8% of participants reported receiving clear discharge instructions. Patient satisfaction was positively associated with the primary 8-item discharge-planning score (Spearman's *ρ* = 0.633, 95% CI: 0.468–0.756, *p* < 0.001). Residence was not statistically significantly associated with discharge-planning quality (B = −1.342, β = −0.193, *p* = 0.081, 95% CI: −2.854 to 0.169), while prior endoscopic experience was associated with higher patient-satisfaction scores (B = 4.066, β = 0.264, *p* = 0.020). Source of information about endoscopy was associated with discharge-planning quality (B = 1.362, β = 0.303, *p* = 0.008), and endoscopic indication was associated with patient satisfaction (B = 1.831, β = 0.241, *p* = 0.030).

**Conclusions:**

Patient satisfaction and discharge-planning quality were positively associated following upper gastrointestinal endoscopy. The descriptive findings identified potential service-improvement priorities, including the provision of written instructions and post-discharge contact information. These observations should not be interpreted as causal implications of the observed association. Given the small, single-center sample, and the exploratory, hypothesis-generating nature of the adjusted analyses, the findings require confirmation in larger, multicenter studies.

## 1 Introduction

Upper gastrointestinal (GI) endoscopy is a commonly used diagnostic and therapeutic procedure for the investigation, surveillance, and management of upper gastrointestinal disorders (1, 2). Upper GI endoscopy is one of the most frequently performed diagnostic procedures worldwide (3, 4). Its use in the United States has also increased across adult age groups, from an estimated 6.1 million Esophagogastroduodenoscopy(EGDs) in 2013 to 7.5 million in 2019 (3). Patient experience is becoming more widely acknowledged as a crucial aspect of endoscopy quality, even though technical quality and procedural efficacy are still crucial elements of endoscopic care (5, 6).

Quality assessment in gastrointestinal endoscopy has traditionally placed a higher priority on technical and procedural performance, but modern quality frameworks, such as the performance measures of the European Society of Gastrointestinal Endoscopy (ESGE) and the quality indicators created by the American Society for Gastrointestinal Endoscopy (ASGE) and the American College of Gastroenterology (ACG), now incorporate patient experience (3, 7).‏ Because it offers a patient-reported viewpoint on the quality of care received across the endoscopic route, patient experience has in particular become a significant quality metric in gastrointestinal endoscopy (3, 5, 6).

Patient experience in gastrointestinal endoscopy is multifaceted, encompassing anxiety and expectations, knowledge and communication, dignity, choice and control, and comfort throughout the endoscopic pathway in addition to procedural comfort. It may also include waiting time, the clarity and adequacy of information provide, instructions with health care professionals, and patients understanding pf postoperative procedure instruction The Group Health Association of America-9 (GHAA-9) and its modified version (mGHAA-9), the Gastrointestinal Endoscopy Satisfaction Questionnaire (GESQ), the Comprehensive Endoscopy Satisfaction Tool (CEST), and the Endoscopy Patient-Reported Experience Measure (ENDOPREM) and the Canadian Satisfaction with Quality of Periprocedural Care (CSSQP) are a few patient-reported tools that have been developed to evaluate various aspects of endoscopy-related patient experience and satisfaction (5, 6). These tools vary in terms of their conceptual scope, the stages of care they evaluate, and their psychometric qualities, such as comprehensiveness, validity, and reliability. According to a recent systematic study, the CEST performed better psychometrically whereas ENDOPREM offered more conceptual coverage. Nevertheless, no single instrument achieved optimal performance in terms of both conceptual coverage and psychometric features (8). The mGHAA-9 was selected because the primary objective of this study was to assess overall patient satisfaction specifically after upper gastrointestinal endoscopy, rather than to capture the full range of periprocedural experiences. Accordingly, its scope was considered appropriate for the study outcome.

Patient satisfaction is a significant patient-centered outcome in gastrointestinal endoscopy and offers a viewpoint on the standard of care from the patient's perspective (9). Previous research has demonstrated that several modifiable aspects of care, such as waiting times, healthcare providers' explanations and communication, staff demeanor, procedural discomfort, and the general handling of patients' needs, may have an impact on patients' satisfaction with endoscopic procedures (10–12). A popular patient satisfaction tool in endoscopy, the modified Group Health Association of America-9 (mGHAA-9) evaluates important aspects of the patient experience, such as waiting time, staff and physician demeanor, medical competence, and Procedural explanation (11).

However, the discharge phase of the endoscopic pathway has received very little empirical attention, despite the increasing amount of data on patient satisfaction and its modifiable variables. Discharge planning is an important component of care following upper gastrointestinal endoscopy since patients need the right information and direction to recover after leaving the endoscopic facility. Following an upper gastrointestinal endoscopy, patients should get clear verbal and written instructions that include guidance on when, where, and how to seek medical assistance if necessary. Before discharge, these guidelines also advise giving patients a summary of the endoscopic results and important recommendations (13). Therefore, good discharge communication may help patients comprehend post-procedure treatment and promote continuity of care following endoscopy (3, 13).‏ Inadequate discharge communication can jeopardize patient safety and continuity of care by causing misunderstandings and insufficient follow-up actions, such as failing to identify alarming symptoms, using medications inappropriately, missing follow-up appointments, and failing to act on significant pathology or biopsy results (7, 14, 15).

Discharge-planning quality has received very little attention as a separate component of post-procedure care, despite the growing recognition of patient experience as a significant quality marker in gastrointestinal endoscopy and the acknowledged vulnerability of the discharge transition, especially in the context of sedated day-case procedures. Discharge signifies the change from post-procedure clinical supervision to at-home care, where patients and, if relevant, their caregivers are in charge of adhering to post-procedure instructions. Clear discharge communication is particularly important during this transition. Current quality standards recommend providing verbal and written instructions before discharge, including information about post-procedure care, potential complications, when and where to seek medical assistance, and follow-up arrangements (13, 14). Patient satisfaction is a crucial measure of gastrointestinal endoscopy quality, particularly upper gastrointestinal endoscopy (3). However, there is limited evidence on the effectiveness of discharge planning following upper gastrointestinal endoscopy and how it relates to patient satisfaction, especially in healthcare settings where Arabic is spoken. This disparity emphasizes the need to investigate whether patient satisfaction after upper gastrointestinal endoscopy is related to the quality of discharge planning. Thus, this study evaluated the quality of discharge planning and patient satisfaction after upper gastrointestinal endoscopy and how it relates to patient satisfaction, especially in healthcare settings where Arabic is spoken. This disparity emphasizes the need to investigate whether patient satisfaction after upper gastrointestinal endoscopy is related to the quality of discharge planning. Thus, this study evaluated the quality of discharge planning and patient satisfaction after upper gastrointestinal endoscopy and investigated the relationship between these outcomes.

### 1.1 Study aim and hypotheses

This study aimed to assess patient satisfaction and discharge-planning quality following upper gastrointestinal endoscopy and to examine the association between them.

The objectives were to

1. Assess patient satisfaction following upper gastrointestinal endoscopy
2. Assess quality of discharge planning following upper gastrointestinal endoscopy
3. Examine the association between patient satisfaction and quality of discharge planning following upper gastrointestinal endoscopy.

We hypothesize that patient satisfaction would be positively associated with discharge planning quality following upper gastrointestinal endoscopy. The hypothesis concerns a cross-sectional relationship only; no directed or causative hypothesis was investigated. Secondary, exploratory, hypothesis-generating multivariable analyzes were carried out.

## 2 Materials and methods

### 2.1 Study design and setting

A single-center cross-sectional study was conducted at the Digestive Endoscopy Service of Qassim University Medical City, Saudi Arabia. Data were collected from 1 April 2024 to 31 October 2024. The study was reported in accordance with the Strengthening the Reporting of Observational Studies in Epidemiology (STROBE) guidelines for cross-sectional studies, as documented in Supplementary File S1 (16). The Digestive Endoscopy Service provided advanced procedures (ERCP, EUS, and EMR), colonoscopies, and EGDs. During the study period, 85 patients underwent upper gastrointestinal endoscopy. Weekly procedure volume varied, and some weeks had no procedures. The head/charge nurse assigns nurses based on procedure complexity, patient acuity, and competency using lean and acuity-based approaches. Endoscopy nurses, gastroenterologists, anesthesiologists, anesthetic technicians, educators, and support personnel make up the multidisciplinary team. Per institutional policy, only an anesthesiologist may provide sedation/anesthesia, including monitored anesthesia care and moderate sedation when clinically necessary. Following treatments, patients are observed while they recover until they receive a physician discharge order and they achieve an Aldrete score of 9–10/10. Standard discharge includes the endoscopy report, discharge summary, and verbal instructions together with written or QR code-based instructions provided by doctors, nurses, and the patient-family educator in both Arabic and English, covering post-procedure care, follow-up, medications, potential adverse events, physician recommendations, and emergency contacts.

### 2.2 Participants and eligibility

Adult patients undergoing upper gastrointestinal endoscopy during the study period were considered eligible for participation. Participants were recruited using a convenience sampling method. Patients were included if they were aged 18-90 years and had undergone upper GI endoscopy during the study period. Patients were excluded if they were unable to understand the study information and complete the online post-procedure questionnaire because of significant visual or communication limitations that prevented them from completing the questionnaire, or if they were under legal guardianship. Among the 85 patients who underwent upper gastrointestinal endoscopy during the study period, five were excluded because they were younger than 18 years. Of the 80 eligible patients who were approached, six declined participations and 74 submitted complete electronic questionnaires, all of which were included in the final analysis. The electronic platform retained submitted questionnaires but did not provide a separate recoverable count of questionnaires that were opened but not submitted; therefore, this number could not be determined retrospectively. The completion proportion among eligible patients approached was 92.5% (74/80). The participant recruitment and selection process is shown in Figure 1. Because the electronic form required completion of all items before submission, no item-level missing data were present among the 74 submitted questionnaires.

**Figure 1:**
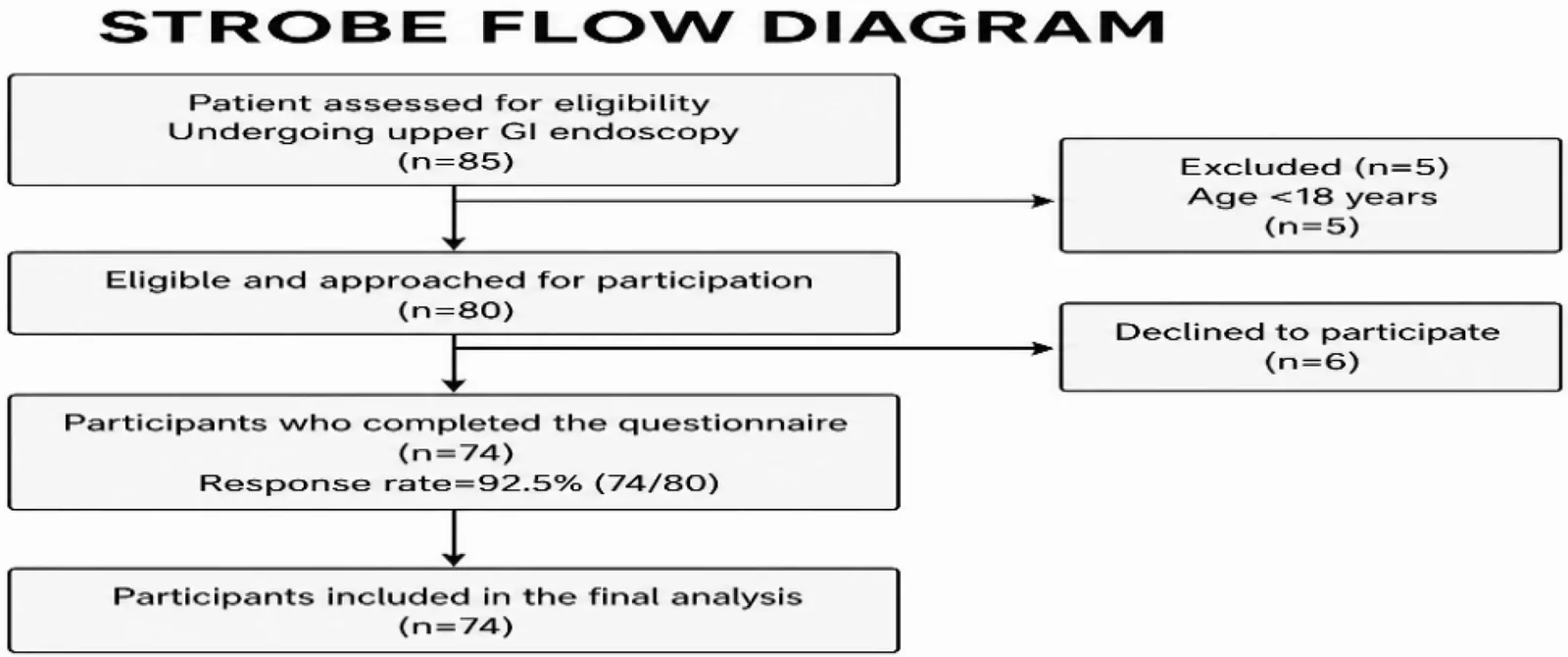
Study participant recruitment flowchart.

### 2.2 Sample size

The original *a priori* sample-size calculation was conducted for the descriptive estimation of a single population proportion rather than for the primary correlational analysis. Using the standard formula *n* = Z^2^P(1−P)/d^2^ (17), with Z = 1.96, *P* = 0.9514 based on a previously published estimate (18), and d = 0.05, the estimated sample size was 71.05 and was rounded up to 72 participants. A total of 74 participants were included in the final analysis. Because *P* ± d = 0.9514 ± 0.05 yields a nominal interval from 0.9014 to 1.0014, exceeding the upper boundary of 1.0, this calculation is reported only as the original descriptive planning rationale and not as a correlation-based sample-size justification. For the primary correlational objective, a sensitivity power analysis was conducted at *n* = 74. Based on published methods for correlation coefficients, including Spearman's correlation (19), a two-sided *α* = 0.05 and noncentral-t approximation with 72 degrees of freedom provided approximately 80% power to detect *ρ* = 0.317, 90% power to detect *ρ* **=** **0.361**,and 95% power to detect *ρ* = 0.396. These values are approximate sensitivity benchmarks because the primary analysis used Spearman's rank correlation and the study was not prospectively powered using an anticipated correlation coefficient.‏ The multivariable regression analyses were not part of the original sample-size calculation and were not prospectively powered. They were therefore conducted and reported as secondary, exploratory, hypothesis generating analyses. the adjusted association should not be interpreted as established independent determinants or predictors and require confirmation in larger, independent samples

### 2.3 Instruments

A self-administered electronic questionnaire was used to assess patient satisfaction and discharge planning quality following upper GI endoscopy. The questionnaire comprised four parts.

#### 2.3.1 Part I: participant information and consent

Provided information about the study objectives, voluntary nature of participation, confidentiality, and eligibility requirements.‏ Informed consent was obtained before participants completed the questionnaire.

#### 2.3.2 Part II: clinical and sociodemographic data

Including age, sex, smoking and alcohol use, marital status, educational attainment, employment status, income level, place of residence, endoscopy information source, chronic illness, prior endoscopy experience, and the indication for upper GI endoscopy as diagnostic/clinical indication, surveillance, or other/unspecified indication based on the reported reason for the procedure, as well as the duration of stay in the endoscopy unit, defined as the total time from arrival to discharge, including preparation, waiting, the procedure, and recovery.‏ For analysis, age was categorized as <30, 30 to <40, 40 to <50, and ≥50 years, while, duration of stay was categorized into three mutually exclusive groups: < 60 min, 60 to 120 min, and >120 min.

#### 2.3.3 Part III: patient satisfaction with upper gastrointestinal endoscopy

The questionnaire used in this study was based on the modified Group Health Association of America-9 (mGHAA-9) instrument, which has been used to assess patient satisfaction (20–22). Patient satisfaction was assessed using the English version of the mGHAA-9 questionnaire, which has been used to assess patient satisfaction with gastrointestinal endoscopy. The questionnaire was translated from English into Arabic. The instrument comprised seven core satisfaction items addressing appointment waiting time at the endoscopy facility, waiting time on the day of the procedure, the physician's interpersonal manner, the manner of nurses and support staff, the adequacy of procedural explanation, procedural discomfort, and the overall visit experience. Each core item was rated on a five-point Likert scale ranging from 1(poor) to 5 (excellent), yielding a total satisfaction score ranging from 7 to 35. The Arabic response options corresponded to poor, fair, good, very good, and excellent, respectively. The response-category order and coding were verified against the electronic questionnaire and the raw dataset, and no data-entry or category-ordering errors were identified. The seven items were treated as ordinal responses, and the total satisfaction score was interpreted as a descriptive composite rather than as a strictly equal-interval measure. Two additional yes/no questions assessed the participants' willingness to undergo the procedure again with the same physician and at the same facility. These two questions were analyzed separately and were not included in the total satisfaction score.‏

#### 2.3.4 Part IV: discharge-planning quality

A preliminary, study-specific questionnaire was developed to evaluate the quality of discharge planning after upper gastrointestinal endoscopy. Based on the literature analysis, pertinent measuring instruments, clinical considerations, and established quality indicators for post-endoscopy care, the study team created the questionnaire items. The questionnaire items were not created by the patients. To find pertinent discharge-planning domains, general discharge-related instruments such as the Readiness for Hospital Discharge Scale (RHDS) and the Quality of Discharge Teaching Scale (QDTS) were examined. Additionally, in order to identify elements of the patient experience pertinent to endoscopic care, endoscopy-specific patient-reported experience measures such as CEST, ENDOPREM, and CSSQP were taken into consideration. Together with clinical considerations and recognized quality indicators for gastrointestinal endoscopic procedures, such as post-procedure patient instructions, the discovered themes were tailored to the particular setting of post-procedure discharge following upper gastrointestinal endoscopy (7, 23).‏

The discharge-planning instrument comprised 10 items representing distinct components of discharge care. These included: clarity of discharge instructions, provision of written instructions, comprehension of written instructions, information about potential complications, instructions for returning to regular activities, medication-related information, dietary advice, availability of contact information for questions or concerns after discharge, satisfaction with discharge information, and overall effectiveness of the discharge plan. The eight factual items constitute the primary 8-item factual discharge-planning index. The two perception-based items were not included in the primary 8-item outcome; they were retained for the 10-item sensitivity analysis. The total discharge-planning quality score was calculated by summing the item scores, with higher scores indicating better discharge-planning quality. For the primary 8-item factual index, the item scores were summed to produce an additive score, with higher scores indicating that more discharge-planning components were reported as provided or understood. The theoretical score for the primary 8-item index ranged from 0 to 9, whereas the observed score range in the study sample was 1 to 9. Depending on the type of inquiry, we used several response formats. One item used a three-point response scale (Yes, fully = 2; Yes, partially = 1; No = 0), and seven items were scored dichotomously (Yes = 1, No = 0). Only participants who reported getting written instructions (*n* = 47) were eligible to use the item measuring comprehension of written instructions. Instead of being marked as “No,” this question was categorized as not applicable because the remaining 27 participants did not receive written instructions. The comprehension item was not included in the overall score or the applicable maximum score for these participants. The items represented distinct facets of discharge care, including information provision, comprehension, recovery instructions, medication and dietary guidance, post-discharge contact information, and immediate patient perceptions. For the primary 8-item factual index, the applicable item scores were added together. Two alternative scoring specifications were examined in sensitivity analyses. The 9-item score excluded the conditional comprehension item and retained the other nine items, whereas the 10-item score included all original questionnaire items, including the two perception-based items. The results of both sensitivity analyses are reported in Supplementary Table S3. For the 9-item score, the maximum score was 15. For the 10-item score, the maximum score was 17 when all 10 items were applicable and 15 when the comprehension item was not applicable because written instructions had not been provided. For descriptive interpretation, each composite score was converted to percentage scores.

### 2.4 Translation of the questionnaire

The modified Group Health Association of America-9 (mGHAA-9) questionnaire and the study-specific Discharge-Planning Quality questionnaire were translated from English to Arabic using the standard forward–backward translation procedure. During the forward–translation phase, two bilingual translators independently translated each questionnaire into Arabic. The two Arabic versions of each questionnaire were compared and reconciled to produce one preliminary Arabic version of each questionnaire. During the backward translation phase, an independent translator who was not involved in the forward translation translated each preliminary Arabic version back into English. Semantic and conceptual equivalence were evaluated by comparing the back-translated versions with the original English versions to find differences. The translation team and expert panel deliberated over any differences until a decision was made.‏ No formal cognitive debriefing was conducted as a separate procedure; however, comprehensibility and response-process issues were assessed during the expert review and pilot testing.

### 2.5 Content validity, pilot testing, and assessment of internal consistency

Content validity was evaluated using a methodical expert-review procedure. Seven experts with backgrounds in public health, nursing, and endoscopic practice independently reviewed the translated Arabic versions of both instruments. The experts assessed the items' clarity, comprehensiveness, and relevance in addition to the electronic format's acceptability and suitability for the study's objectives and target audience. The content validity ratio (CVR), the scale-level content validity index based on the average method (S-CVI/Ave), and the item-level content validity index (I-CVI) were used to measure content validity. Every item showed an I-CVI of 1.00 for both clarity and relevance. Based on the experts' final essentiality evaluations, the CVR was 1.00 for every item, and the S-CVI/Ave was 1.00 for relevance and 1.00 for clarity. Ten participants took part in a pilot test after the expert review. They were recruited separately from the main study sample, excluded from the main study and final analysis, and were not invited to participate again. The pilot test was conducted to assess the feasibility and clarity of the instruments and was not used to calculate the reported reliability estimate. Prior to the primary data collection, the instruments were finalized, and participant feedback was reviewed. The discharge-planning instrument was conceptualized as a formative index comprising distinct elements of discharge care rather than as a reflective scale measuring a single latent construct. Accordingly, internal consistency was not considered the primary psychometric property of this index. Its evaluation focused on expert-based content validity and a prespecified scoring rule, including explicit handling of the conditional comprehension item. Cronbach's alpha was retained only for the Patient Satisfaction Scale, for which internal consistency was assessed in the full primary study sample (*N* = 74). The Patient Satisfaction Scale showed excellent internal consistency (Cronbach's *α* = 0.922; 95% CI: 0.886–0.944).

### 2.6 Data collection procedure

The data were gathered by a member of the research team. In order to identify possibly eligible patients, the study team worked with the chief nurses of the pertinent departments at Qassim University Medical City prior to participant recruitment. Following upper gastrointestinal endoscopy and prior to discharge, eligible patients were contacted. Patients were deemed ready to participate if they were cognizant, competent to give informed consent, clinically stable, and alert. Data collection was conducted only after participants had fully recovered from sedation, were alert and clinically stable, and had achieved an Aldrete score of 9–10/10. All verbal and written discharge instructions were provided before questionnaire completion, and participants were confirmed to be sufficiently aware to understand the study information, provide informed consent, and complete the questionnaire. Participants used a web link or QR code to access the self-administered electronic questionnaire after giving their consent. Using an electronic device, the questionnaire took 5 to ten minutes to complete. The electronic form could not be submitted until all needed questionnaire items were filled out. Only participants who had trouble accessing or navigating the electronic questionnaire received technical assistance from a member of the study team. Help was restricted to using the computer platform and accessing the questionnaire; it did not include elucidating the meaning of particular questions, suggesting answers, or swaying participants' responses. No names or other personally identifiable information was gathered from the questionnaire. Upon submission, answers were automatically added to an electronic database that was password-protected. Only approved members of the research team had access to the database.

### 2.7 Ethical approval and consent to participate

The Research Ethics Committee of the Deanship of Scientific Research at Qassim University approved this study on 2 January 2024) approval No. 24-71-02), before 1 April 2024. Institutional permission was also obtained from authorized authorities at Qassim University Medical City. Every participant received information on the study's goals, methods, voluntary nature, possible risks and rewards, and their freedom to leave the study at any moment without incurring any fees. Before accessing the questionnaire, each participant reviewed the study information and provided electronic informed consent by selecting the agreement option at the beginning of the online survey. The questionnaire did not request participant names, medical record numbers, telephone numbers, email addresses, or other direct personal identifiers; therefore, responses were collected anonymously. The data were stored on a password–protected platform accessible only to authorized members of the research team.

### 2.8 Statistical analysis of the data

IBM SPSS software package version 28.0 (IBM Corp., Armonk, NY, USA) was used to analyze the data that were fed into the computer. Numbers and percentages were used to characterize qualitative data. Quantitative variables were reported using range (minimum and maximum), mean, standard deviation, median, and interquartile range (IQR), when appropriate, and the Kolmogorov–Smirnov test was employed to confirm that the distribution was normal. The acquired results were evaluated for significance at the 5% level. For binary categorical variables, scores were compared using the Mann–Whitney U test; for categorical variables with more than two levels, the Kruskal–Wallis test was utilized. When necessary, *post hoc* pairwise comparisons were performed using Dunn's test. Spearman's rank correlation coefficient was used to assess the association between patient satisfaction and discharge-planning quality because the score distributions were non-normally distributed. The discharge-planning score was treated as a formative additive index, and its interpretation was based on the prespecified scoring rule and expert-based content validity rather than on internal consistency. Two separate secondary, exploratory, hypothesis –generating multivariable linear regression models were fitted: one for patient satisfaction and one for discharge-planning quality. Three candidate predictors were considered for patient satisfaction and five for discharge-planning quality—age, marital status, educational level, residence, and source of information about endoscopy**—**based on clinical relevance and preliminary unadjusted analyses using a data-driven threshold of *p* < 0.10. These multivariable analyzes were carried out as secondary, exploratory, hypothesis-generating analyzes and were not prospectively powered due to the small sample size (*n* = 74). The adjusted associations are therefore not interpreted as established independent determinants or predictors and require validation in larger, independent samples. Indications were classified according to the documented clinical purpose of the procedure: surveillance for planned follow-up of a previously diagnosed condition or lesion; diagnostic/clinical indications for symptom evaluation, suspected disease, diagnostic assessment, or risk-based screening without an established diagnosis; and other/unspecified when the indication was unclear. Because of the cross-sectional design and the exploratory predictor-selection process, these models were not intended to establish causal relationships. The final dataset contained no missing responses. For participants who did not get written instructions, the written-instruction comprehension item was deemed inapplicable. No missing data were identified among the 74 participants; therefore, no imputation was required.‏ The primary analysis used the 8-item factual discharge-planning score. Two sensitivity analyses were also conducted using the 9-item and 10-item discharge-planning scores. The primary 8-item analysis is presented in Supplementary Table S2, and the 9-item and 10-item sensitivity analyses are presented in Supplementary Table S3. We performed residual diagnostics for both linear regression models using histograms, normal P–P plots, and scatterplots of standardized residuals against adjusted predicted values. Influence diagnostics included Mahalanobis distance, Cook's distance, and centered leverage values. The corresponding plots are provided in Supplementary Table S4 and S5.

## 3 Results

The baseline characteristics of the study participants (*n* = 74) are presented in Table 1. Most participants were Saudi nationals and females with higher education, living in urban areas, and reporting sufficient income. Exactly half of the participants were married (50.0%) and did not smoke or consume alcohol (86.5%). Information regarding endoscopy was primarily obtained from formal healthcare providers (63.5%). Approximately one-third of the participants reported having a chronic illness (33.8%). Diabetes mellitus 15 [60.0%], hypertension 5 [20.0%], and asthma 4 [16.0%] were among the chronic diseases reported. Most participants underwent upper gastrointestinal endoscopy for diagnostic/clinical indications (70.3%), followed by other/unspecified indications) *n* **=** **19, 25.7%**) and surveillance (*n* **=** **3, 4.1%**).‏ The median patient–reported total time spent in the endoscopy unit was 30 min (IQR 30–120). Because the clinical pathway included preparation, waiting, the procedure, recovery to an Aldrete score of 9–10, physician discharge authorization, and discharge instructions, the lower reported durations may reflect underestimation, recall error, or interpretation of the question as referring primarily to procedure duration rather than the complete unit stay.

**Table 1:** Baseline characteristics of the study participants (*N* = 74).

Characteristic	Value
**Age, years**	
<30	31 (41.9)
30 - < 40	9 (12.2)
40- < 50	22 (29.7)
≥50	12 (16.2)
**Min.—Max.**	**18.0- 72.0**
**Mean ± SD**	**35.4** **±** **13.2**
**Median (IQR)**	**33.5 (22.5–45.5)**
**Sex**	
Male	19 (25.7)
Female	55 (74.3)
**Nationality**	
Saudi	55 (74.3)
Non-Saudi	19 (25.7)
**Smoking**	
Yes	9 (12.2)
No	65 (87.8)
**Alcohol**	
Yes	1 (1.4)
No	73 (98.6)
**Marital status**	
Married	37 (50.0)
Single	33 (44.6)
Divorced	4 (5.4)
**Educational level**	
Below secondary	2 (2.7)
Secondary	8 (10.8)
Higher education	64 (86.5)
**Employment status**	
Employed	37 (50.0)
Student	22 (29.7)
Unemployed + Retired	15 (20.3)
**Income level**	
Sufficient	67 (90.5)
Insufficient	7 (9.5)
**Residence**	
Rural	8 (10.8)
Urban	66 (89.2)
**Source of information about endoscopy**	
Informal sources (social media/internet/friends/other)	27 (36.5)
Formal sources (health care providers)	47 (63.5)
**Chronic disease**	
Yes	25 (33.8)
No	49 (66.2)
**Which type of chronic disease do you suffer from?**	
Diabetes mellitus	15 (60.0)
Hypertension	5 (20.0)
Asthma	4 (16.0)
Arthritis	1 (4.0)
**Previous. Endoscopy. Experience**	
No	56 (75.7)
Yes	18 (24.3)
**Indication for upper gastrointestinal endoscopy**	
Diagnostic/clinical indications	52 (70.3)
Surveillance	3 (4.1)
Other/unspecified indications	19 (25.7)
**Total time spent in the endoscopy unit**	
< 60 min	51 (68.9)
60–120 min	18 (24.3)
>120 min	5 (6.8)
**Total time spent in the endoscopy unit**	
Min.—Max.	30–180
Mean ± SD	66 ± 48
Median (IQR)	30 (30–120)

Data are presented as *n* (%), mean ± standard deviation (SD), or median (interquartile range (IQR). Chronic disease type percentages were calculated among participants with chronic disease (*n* = 25); all other percentages were based on the total sample (*N* = 74).

Table 2 shows the distribution of responses to the patient-satisfaction items. The doctor's personal manner had the highest percentage of excellent ratings among the core satisfaction categories (52/74, 70.3%), followed by the manner of the nurses and support staff and the visit's overall assessment (51/74, 68.9% each). Additionally, waiting time for an appointment (46/74, 62.2%), waiting time on the day of the treatment (41/74, 55.4%), comfort during the process (49/74, 66.2%), and adequate explanation (48/74, 64.9%) were all rated as excellent. Regarding the two extra yes/no items, 65 participants (87.8%) said they would repeat endoscopy with the same doctor, and 70 participants (94.6%) said they would repeat the procedure at the same facility.

**Table 2:** Patient-reported satisfaction with upper gastrointestinal endoscopy (*N* = 74).

Panel A. Core patient satisfaction items					
Item	Poor	Fair	Good	Very good	Excellent
Waiting time to obtain an appointment	7 (9.5)	1 (1.4)	15 (20.3)	5 (6.8)	46 (62.2)
Waiting time on procedure day	7 (9.5)	6 (8.1)	15 (20.3)	5 (6.8)	41 (55.4)
Physician's personal manner	0 (0.0)	7 (9.5)	12 (16.2)	3 (4.1)	52 (70.3)
Nurses'/support staff manner	0 (0.0)	6 (8.1)	17 (23.0)	0 (0.0)	51 (68.9)
Adequacy of explanation	1 (1.4)	8 (10.8)	12 (16.2)	5 (6.8)	48 (64.9)
Comfort during procedure	0 (0.0)	5 (6.8)	16 (21.6)	4 (5.4)	49 (66.2)
Overall rating of the visit	0 (0.0)	5 (6.8)	10 (13.5)	8 (10.8)	51 (68.9)

Panel B. Additional yes/no satisfaction items					
Items					No(%)
Would you have the procedure done again by this physician?					
Yes					65 (87.8)
No					9 (12.2)
Would you have the procedure done again at this facility?					
Yes					70 (94.6)
No					4 (5.4)

Data are presented as *n* (%). Higher values indicate greater satisfaction.

Data are presented as *n* (%). Panel A items were rated on a five-point scale ranging from poor to excellent. The two additional yes/no items in Panel B were reported separately and were not included in the calculation of the total patient satisfaction score. Higher rating indicates greater satisfaction.

Discharge planning was also evaluated favorably overall. Clear discharge instructions were reported by 62/74 participants (83.8%).‏ A total of 47/74 participants (63.5%) received written instructions. Among those 47 participants who actually received written instructions, 38 (80.9%) reported complete understanding, 7 (14.9%) partial understanding, and 2 (4.3%) no understanding. Information regarding management of possible complications was reported by 54/74 participants (73.0%), guidance on resumption of normal activities by 65/74 (87.8%), received medication-related information by 59/74 (79.7%), and contact information for post-discharge questions by 36/74 (48.6%) (Table 3).

**Table 3:** Patient-reported quality of discharge planning after upper gastrointestinal endoscopy.

Item	Response category	*n* (%)	Denominator
Provided clear discharge instructions	No	12 (16.2)	74
	Yes	62 (83.8)	74
Given written instructions	No	27 (36.5)	74
	Yes	47 (63.5)	74
Understood written instructions^a^	No	2 (4.3)	47
	Yes, partially	7 (14.9)	47
	Yes, completely	38 (80.9)	47
Informed about what to do in case of complications	No	20 (27.0)	74
	Yes	54 (73.0)	74
Given instructions on resuming normal activities	No	9 (12.2)	74
	Yes	65 (87.8)	74
Received dietary instructions after the procedure	No	45 (60.8)	74
	Yes	29 (39.2)	74
Received medication-related information	No	15 (20.3)	74
	Yes	59 (79.7)	74
Given contact information for post-discharge questions/concerns	No	38 (51.4)	74
	Yes	36 (48.6)	74

Data are presented as *n* (%).

a This item was assessed only among participants who reported receiving written instructions (*n* = 47). The remaining 27 participants didn't receive written instructions; therefore, this item did not apply to them and was excluded from their individual score and relevant maximum possible score.

Table 4 demonstrates that overall patient satisfaction and the quality of discharge planning were both good. The median percentage score was 92.9% (IQR, 46.4–100.0%), while the median overall patient satisfaction score was 33.0 (IQR, 20.0–35.0). For the primary 8-item factual discharge-planning outcome, the median score was 7.0 (IQR, 5.0–8.0), corresponding to a median percentage score of 77.8% (IQR, 55.6–88. 9%). The distributions of the two composite scores are presented in Figure 2, and visual inspection suggested clustering of scores toward the upper end of both scales, particularly for patient satisfaction.‏ The association between the primary 8-item factual discharge-planning percentage score and the patient-satisfaction percentage score was statistically significant (Spearman's *ρ* = 0.633, 95% CI: 0.468–0.756, *p* < 0.001; Supplementary Table S2).

**Table 4:** Overall patient satisfaction and discharge-plan quality following upper GI endoscopy (*N* = 74).

Outcome	Value
**Patient satisfaction total score**	
Min.—Max.	15.0–35.0
Mean ± SD	29.6 ± 6.7
Median (IQR)	33.0 (20.0–35.0)
**Patient satisfaction percentage score**	
Min.—Max.	28.6–100.0
Mean ± SD	80.8 ± 23.8
Median (IQR)	92.9 (46.4–100.0)
**Discharge-plan total score**	
Min.—Max.	1.0–9.0
Mean ± SD	6.20 ± 2.18
Median (IQR)	7.0 (5.0–8.0)
**Discharge-plan percentage score**	
Min.—Max.	11.11–100.0
Mean ± SD	68.9 ± 24.2
Median (IQR)	77.8 (55.6–88.9)

Data are presented as mean ± standard deviation (SD) or median (IQR), as appropriate. The primary discharge-planning outcome was based on the 8-item factual index.

**Figure 2:**
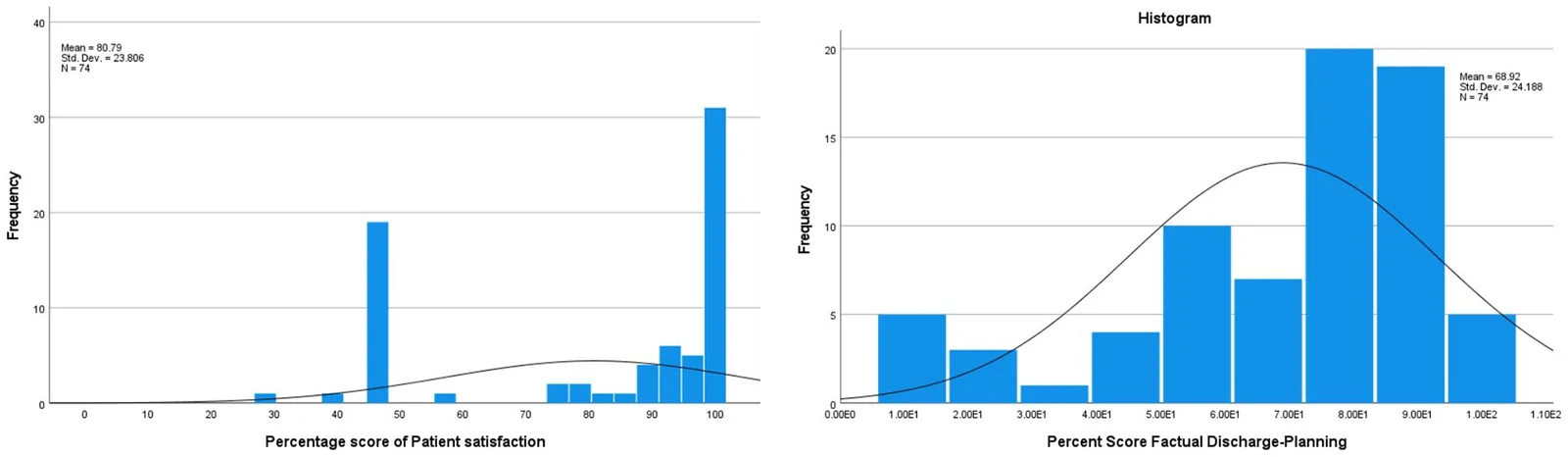
Distribution of patient-satisfaction and discharge-planning quality scores following upper gastrointestinal endoscopy. The distributions of both composite scores were concentrated toward the upper ends of their respective scales, particularly for patient satisfaction.

Table 5 shows a significant positive correlation between patient satisfaction percentage score and primary 8-item factual discharge-planning quality percentage score (Spearman's *ρ* = 0.633, 95% CI: 0.468–0.756, *p* < 0.001). This result supports the hypothesis that patient satisfaction is positively associated with the primary discharge-planning quality outcome. The primary 8-item analysis is presented in Supplementary Table S2, whereas the 9-item and 10-item sensitivity analyses are reported in Supplementary Table S3.

**Table 5:** Correlation between patient-satisfaction percentage score and primary 8-item factual discharge-planning percentage score following upper GI endoscopy (*N* = 74).

Variable pair	Correlation coefficient	95% CI	*p* value
Patient-satisfaction percentage score vs. 8-item factual discharge-planning percentage **score**	Spearman's rho = 0.633	0.468 to 0.756	<0.001

normally distributed. CI indicates the confidence interval.

Table 6 shows the unadjusted analyses; patient-satisfaction scores were significantly associated with smoking habits, and discharge-planning quality scores were significantly associated with age, marital status, educational level (*p* = 0.049), residence, and source of endoscopy information. In the unadjusted analysis, rural participants had higher discharge-planning quality scores than urban participants, with mean scores of 7.8 and 6.0 and median scores of 8.0 and 7.0, respectively (*p* = 0.018).

**Table 6:** Unadjusted associations between sociodemographic and clinical characteristics and total patient satisfaction and discharge-planning quality scores following upper gastrointestinal endoscopy (*N* = 74).

Variable	Category/scale	Total patient satisfaction score				Total discharge-plan score			
		Mean	±SD	Median	p	Mean	±SD	Median	p
Age, years	<30	28.0	7.4	32.0	0.121	5.4	2.7	7.0	0.005
	30 - <40	30.0	6.0	32.0		6.2	1.6	7.0	
	40- <50	33.0	3.6	35.0		7.5	1.2	8.0	
	≥50	27.3	7.6	26.5		5.9	1.2	5.5	
Dunn *post hoc*						P1 = 0.770,p2 = 0.001,p3 = 0.702,p4 = 0.049 ,p5 = 0.585,p6 = 0.005			
Sex	Male	30.8	5.9	33.0	0.645	7.0	1.7	7.0	0.088
	Female	29.2	6.9	33.0		5.9	2.3	7.0	
Nationality	Saudi	29.0	6.8	32.0	0.139	6.1	2.4	7.0	0.810
	Non-Saudi	31.4	6.1	35.0		6.6	1.2	7.0	
Smoking habits	Yes	34.4	1.3	35.0	0.008	7.1	1.2	7.0	0.202
	No	28.9	6.9	33.0		6.1	2.3	7.0	
Marital status	Married	30.6	5.9	33.0	0.422	6.9	1.4	7.0	0.026
	Divorced	31.3	7.5	35.0		7.0	2.0	8.0	
	Single	28.4	7.3	32.0		5.3	2.6	7.0	
Dunn *post hoc*						P1 = 0.651,p2 = 0.012,p4 = 0.114			
Educational level	Below secondary	26.5	9.2	26.5	0.574	5.5	3.5	5.5	0.049
	Secondary	28.5	7.3	31.5		5.1	0.8	5.0	
	Higher education	29.9	6.6	33.5		6.4	2.2	7.0	
Dunn *post hoc*						P1 = 0.396,p2 = 0.729,p4 = 0.014			
Employment status	Employed	29.8	7.0	34.0	0.733	6.6	1.8	7.0	0.432
	Student	28.4	7.0	32.5		5.5	2.8	7.0	
	Unemployed + Retired	31.1	5.1	33.0		6.3	1.8	6.0	
Income level	Sufficient	29.4	6.9	34.0	0.608	6.1	2.2	7.0	0.287
	Insufficient	31.4	2.7	32.0		7.0	1.5	8.0	
Residence	Rural	30.5	6.8	35.0	0.416	7.8	0.7	8.0	0.018
	Urban	29.5	6.7	33.0		6.0	2.2	7.0	
Source of information about endoscopy	Informal sources (social media/internet/friends/other)	28.3	7.1	32.0	0.127	5.1	2.9	6.0	0.012
	Formal sources (health care providers)	30.4	6.4	34.0		6.9	1.3	7.0	
Previous. Endoscopy. Experience	No	28.5	7.1	32.5	0.081	5.9	2.4	7.0	0.153
	Yes	33.1	3.6	34.0		7.0	1.2	7.0	
Chronic disease	Yes	29.4	6.8	33.0	0.797	6.3	1.5	7.0	0.441
	No	30.1	6.5	33.0		6.2	2.5	7.0	
Indication for upper gastrointestinal endoscopy	Diagnostic/clinical indications	28.7	7.1	33.0	0.064	6.2	2.3	7.0	0.426
	Surveillance	26.0	7.9	23.0		3.3	4.0	1.0	
	Other/unspecified indications	32.8	3.8	35.0		6.7	1.2	7.0	
Duration of stay in the endoscopy unit category	≤1 h	29.3	7.0	34.0	0.208	5.9	2.4	7.0	0.075
	1–2 h	30.0	6.5	33.0		6.4	1.6	7.0	
	>2 h	32.0	3.7	34.0		8.0	0.0	8.0	

P, *p* value for Mann–Whitney or Kruskal–Wallis test followed by Dunn's *post hoc* tests with Bonferroni adjustment;.

P1, *p* value for comparing between category a and b,.

P2, *p* value for comparing between category a and c,.

P3, *p* value for comparing between category a and d,.

P4, *p* value for comparing between category b and c,.

P5, *p* value for comparing between category b and d.

P6, *p* value for comparing between category c and d.

B, unstandardized regression coefficient; SE, standard error; β, standardized regression coefficient; CI, confidence interval; LL, lower limit; UL, upper limit; VIF, variance inflation factor; R^2^, coefficient of determination.

Secondary exploratory multivariable analysis. As shown in Table 7, a secondary, exploratory, hypothesis -generating multivariable linear regression model was fitted to examine factors associated with patient satisfaction, with candidate predictors selected based on clinical relevance and a data-driven screening threshold of *p* < 0.10 in the preliminary unadjusted analyses. The model was statistically significant and explained 19.5% of the variance in total patient satisfaction score (R^2^ = 0.195; adjusted R^2^ = 0.160; F = 5.635; *p* = 0.001). Previous endoscopic experience was statistically associated with a higher patient satisfaction score (B = 4.066; β = 0.264; *p* = 0.020; 95% CI: 0.659–7.473). Indication (other/unspecified indications) was also significantly associated with patient satisfaction (B = 1.831; β = 0.241; *p* = 0.030; 95% CI: 0.182–3.480). In contrast, smoking was not statistically significantly associated with patient satisfaction (B = −3.585; β = −0.185; *p* = 0.103; 95% CI: −7.913–0.742). These adjusted associations as exploratory findings and are not interpreted as established independent determinants or predictors of patient satisfaction. The adjusted estimates and corresponding 95% confidence intervals from the exploratory multivariable linear regression models are presented in Figure 3. Residual plots for the patient-satisfaction regression model, including the histogram, normal P–P plot, and scatterplot of standardized residuals against adjusted predicted values, are presented in Supplementary Figure S1. The corresponding residual and influence diagnostics, including Mahalanobis distance, Cook's distance, and centered leverage values, are reported in Supplementary Table S4.

**Table 7:** Multivariable linear regression analysis of factors associated with total patient satisfaction scores following upper GI endoscopy.

Variable	B	SE	Beta	t	P	95% CI of B		Tolerance	VIF
						LL	UL		
(Constant)	32.473	4.623		7.024	0.000	23.253	41.693		
Smoking (Noo)	−3.585	2.170	−0.185	−1.653	0.103	−7.913	0.742	0.917	1.091
Previous. Endoscopy. Experience	4.066	1.708	0.264	2.380	0.020	0.659	7.473	0.939	1.065
Indication (Other/unspecified indications)	1.831	0.827	0.241	2.215	0.030	0.182	3.480	0.970	1.031

R^2^ = 0.195, adjusted R^2^ = 0.160, F = 5.635, *p* = 0.001.

F,p, f and *p* values for the model; R^2^, Coefficient of determination.

B, Unstandardized Coefficients; SE, Estimates Standard error.

Beta, Standardized Coefficients; t, t-test of significance.

CI, Confidence interval; LL, Lower limit; UL, Upper Limit.

**Figure 3:**
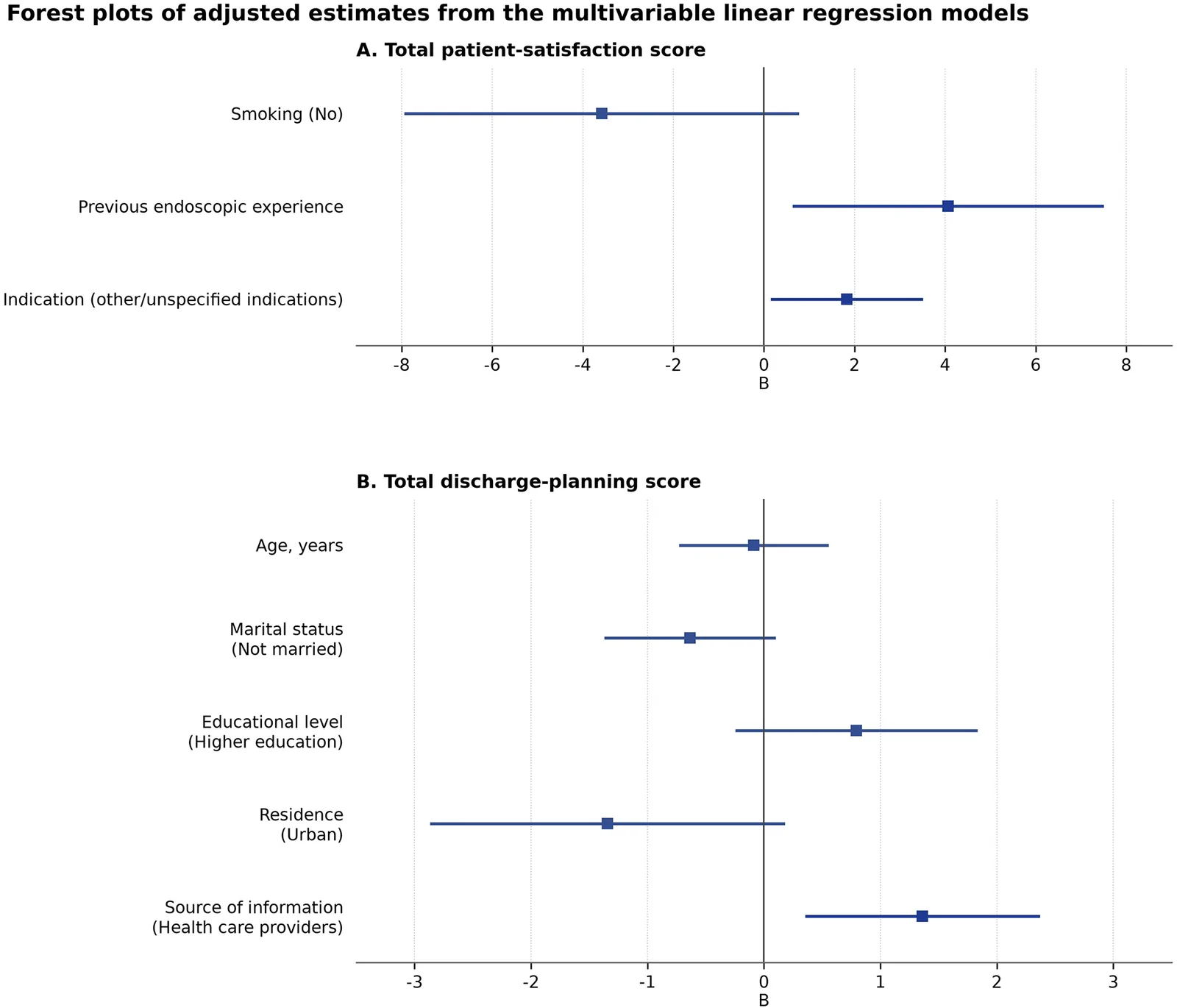
Forest plots of adjusted estimates from **the exploratory** multivariable linear regression models examining factors associated with patient satisfaction and discharge-planning quality following upper gastrointestinal endoscopy. **(A)** Total discharge-planning score. **(B)** Total patient-satisfaction score. Points represent adjusted regression coefficients **(B)**, and horizontal lines represent the corresponding 95% confidence intervals.

A secondary exploratory multivariable analysis. An exploratory multivariable linear regression model was fitted as a secondary, hypothesis –generating analysis to examine factors associated with discharge-planning quality, with candidate predictors selected based on clinical relevance and a data-driven screening threshold of *p* < 0.10 in the preliminary unadjusted analyses. This model was not the primary analysis and was not prospectively powered. The discharge-planning quality multivariable linear regression model explained 28.2% of the variance in the primary 8-item factual discharge-planning score (R^2^ = 0.282; adjusted R^2^ = 0.229) and was statistically significant (**F** **=** **5.341, *p*** **<** **0.001**). Residual diagnostics did not show a major funnel-shaped pattern or extreme residuals. However, some deviation from normality was observed, particularly for the patient-satisfaction model. The regression findings were therefore interpreted as exploratory associations. The corresponding residual plots are presented in Supplementary Figure S2, and the residual and influence diagnostics are presented in Supplementary Table S5. The maximum Mahalanobis distance was 24.546 in the five-predictor model, suggesting that one observation warranted further assessment for potential multivariate influence; the maximum Cook's distance was 0.371, and the maximum centered leverage value was 0.336. In unadjusted analyses, discharge-planning quality was significantly associated with age, marital status, educational level, residence, and source of endoscopy information. After adjustment, only obtaining endoscopy information from healthcare providers remained significantly associated with a higher discharge-planning quality score (B = 1.362, β = 0.303, *p* = 0.008, 95% CI: 0.367–2.357) (Table 8). As with the satisfaction model, this coefficient is an adjusted association from an exploratory model, not evidence that the source of endoscopy information is an independent determinants or predictor of discharge—planning quality, and it requires confirmation in larger independent samples.

**Table 8:** Multivariable linear regression analysis of factors associated with total discharge-planning quality score following upper gastrointestinal endoscopy.

Variable	B	SE	Beta	t	p	95% CI of B		Tolerance	VIF
						LL	UL		
(Constant)	5.688	2.291		2.482	0.016	1.116	10.260		
Age, years	−0.087	0.317	0.046	0.276	0.784	−0.719	0.544	0.372	2.686
Marital status (Not married)	−0.635	0.363	−0.285	−1.750	0.085	−1.360	0.089	0.397	2.518
Educational level (Higher education)	0.795	0.515	0.160	1.544	0.127	−0.232	1.822	0.981	1.019
Residence (Urban)	−1.342	0.757	−0.193	−1.773	0.081	−2.854	0.169	0.893	1.120
Source of information about endoscopy (health care providers)	1.362	0.499	0.303	2.732	0.008	0.367	2.357	0.857	1.167

R^2^ = 0.282, adjusted R^2^ = 0.229, F = 5.341, *p* < 0.001.

F,p, f and *p* values for the model; R^2^, Coefficient of determination.

B, Unstandardized Coefficients; SE, Estimates Standard error.

Beta, Standardized Coefficients; t, t-test of significance.

CI, Confidence interval; LL, Lower limit; UL, Upper Limit.

## 4 Discussion

Patient satisfaction after upper gastrointestinal endoscopy was generally favorable in the current study. The median percentage score was 92.9% (IQR, 46.4–100.0%) while the median overall satisfaction score was 33.0 (IQR, 20.0–35.0). Comfort during the process (66.2%), adequate explanation (64.9%), and the doctor's personal manner (70.3% rated it excellent), nurses' and support staff's manner, and the visit's overall assessment (68.9% each) received the highest ratings. Furthermore, the majority of patients said they would repeat endoscopy at the same institution (94.6%) and with the same doctor (87.8%). These findings indicate a generally favorable patient-reported experience, particularly with respect to interpersonal care, communication, and procedural comfort; however, they do not establish that these factors caused higher satisfaction.

This result is consistent with other studies that show how crucial interpersonal and communication-related elements of endoscopic care are to patient satisfaction. According to a recent study involving 191 patients undergoing endoscopic procedures, patients' satisfaction with the information they received both before and after the procedure, as well as their opinions of the doctors and degree of trust, were all positively correlated with their overall experience (12). Additionally, waiting time received positive ratings in the current study: 55.4% of participants assessed the waiting time on the day of the treatment as excellent, and 62.2% rated the waiting time for an appointment as excellent. Waiting time should not be construed as a factor associated with either outcome in this sample since it was not linked to either patient satisfaction or the quality of discharge planning in the current study. In a recent study evaluating patient satisfaction in a digestive endoscopy service, waiting periods earned comparatively lower ratings, whereas nursing care, medical care, procedural explanations, and procedural comfort were among the highest-rated elements (24). Overall, these results are consistent with the current study, which showed that physician and nursing staff manner, procedural comfort, and the adequacy of explanation

This study's comparatively positive procedure comfort rating is also clinically significant. In endoscopy, comfort is becoming more widely acknowledged as a quality measure and a significant part of the patient experience rather than just an incidental procedure outcome (6). 66.2% of subjects in the current study gave comfort an excellent rating, indicating a generally favorable patient-reported experience of procedural comfort. However, as about one-third of participants did not choose the highest rating, this finding should not be taken as suggesting that discomfort was insignificant. Variations in sedation techniques, patient anxiety, procedure technique, and personal pain perception may cause variations in comfort and satisfaction among endoscopic populations. These results are also in line with those of (25), who found associations between waiting times, the demeanor of doctors and staff, the quality of explanations given both before and after the treatment, and pain or discomfort and patient satisfaction. These findings support the relevance of these factors as components of patient experience but do not establish that modifying any single factor would necessarily increase satisfaction.

Discharge-planning quality in the current study was generally good, with a median 8-item primary 8-item factual discharge-planning score of 7.0 (IQR, 5.0–8.0), corresponding to a median percentage score of 77.8% (IQR, 55.6–88.9%). While information about returning to normal activities, medications, and potential risks was given very often, the majority of patients reported getting clear discharge instructions. These findings indicate that several core discharge components were commonly reported, although the completeness of discharge planning was not uniform across all items. This generally positive pattern indicates that many patients received sufficient post-endoscopy education. The results align with existing quality frameworks, which acknowledge post-procedural safety and patient experience as critical elements of superior upper gastrointestinal endoscopy (3, 6).‏

The findings, however, also highlighted several deficiencies in discharge planning. Specifically, dietary recommendations were reported less frequently than other discharge components, and fewer than half of the participants reported receiving contact information for post-discharge questions or concerns. This implies that not all components of discharge planning were regularly provided, despite the overall positive score. Patients may experience symptoms after leaving the endoscopy unit and need to know when and where to seek help, so having clear plans for post-discharge communication is very crucial. A recent systematic study also emphasized the need for safe and well-defined discharge procedures and the variation in outpatient discharge practices after gastrointestinal endoscopy (26).‏ Effective communication between healthcare providers and patients prior to release may contribute to the relatively favorable discharge-planning quality found in this study. This is in line with data from a 2024 study that showed a positive correlation between overall patient experience and satisfaction with information given both prior to and following endoscopy (12).‏ The gaps in dietary instructions and post-discharge contact information represent particularly actionable areas for service improvement. Standardizing dietary advice and providing clear contact information for post-discharge questions or concerns may strengthen the completeness and continuity of discharge planning. However, the current results point to ways to improve discharge planning even further, especially by providing clear post-discharge contact information and standardized written instructions that could improve the comprehensiveness and continuity of care (26).

Patient satisfaction and the quality of discharge planning after upper gastrointestinal endoscopy were found to be significantly positively correlated in the primary analysis based on the 8-item factual discharge-planning index (Spearman's *ρ*= 0.633, 95% CI: 0.468–0.756, *p* < 0.001). Thus, the research hypothesis was supported by a statistically significant positive cross-sectional link between the two measures; however, neither causality nor the direction of the relationship was established by this finding. The 8-item analysis was considered the primary analysis because it focused on factual discharge-planning components and minimized potential overlap with patient-perception items. Across the sensitivity analyses, Spearman's *ρ* slightly declined from 0.630 to 0.595 while maintaining the same direction and significance. These results suggest that the conditional written-instruction comprehension item was not the only factor contributing to the observed association.‏ Thus, the findings remain informative regarding patient-reported discharge communication at the point of discharge, but should not be interpreted as evidence of sustained post-discharge recall or adherence. This implies that patients who had a better overall endoscopic experience also tended to have more positive perceptions of their discharge planning. One explanation could be that discharge planning is a component of the whole patient experience, especially when it comes to giving clear instructions, assurances, and information regarding post-procedural care. This interpretation is consistent with previous research emphasizing the importance of information provision, patient–centered communication, and trust in shaping patients' experiences of endoscopy (12). Individualized discharge planning may enhance patient satisfaction, according to data from a Cochrane systematic review; however, the evidence's degree of certainty is low (27).‏ Recent research has also shown that outpatient discharge practices vary in the particular setting of gastrointestinal endoscopy, underscoring the significance of well-defined discharge procedures for safe post-procedural care (26).‏ The observed association should therefore be interpreted as a patient-reported cross-sectional relationship rather than evidence of a treatment effect.

The majority of sociodemographic and clinical traits were not significantly associated with overall patient satisfaction after upper gastrointestinal endoscopy in the unadjusted analysis. Participants who reported smoking had higher mean satisfaction scores than those who did not (34.4 ± 1.3 vs. 28.9 ± 6.9; *p* = 0.008). Nevertheless, after adjustment in the multivariable model, this association was not statistically significant (B = −3.585, β = −0.185, *p* = 0.103, 95% CI: −7.913 to 0.742), indicating that the unadjusted association may have reflected other factors. In the exploratory adjusted model, prior endoscopic experience was associated with higher patient satisfaction (B = 4.066, β = 0.264, *p* = 0.020, 95% CI: 0.659–7.473) and endoscopic indication was also associated with patient satisfaction (B = 1.831, β = 0.241, *p* = 0.030, 95% CI: 0.182–3.480). These adjusted associations are reported as exploratory findings and should not be interpreted as established independent determinants or predictors of patient satisfaction; they require confirmation in larger, independent samples. Because anxiety and patient expectations were not directly measured, this link should not be construed as causative given the cross-sectional methodology, and its underlying processes are yet unknown. Prior research has demonstrated that the quality of the care experience—including the endoscopist's interpersonal demeanor and technical proficiency, nursing care, procedure communication, and pain or discomfort—influences patient satisfaction with gastrointestinal endoscopy (28, 29). The multivariable model was statistically significant (R^2^ = 0.195, adjusted R^2^ = 0.160, F = 5.635, *p* = 0.001) and accounted for 19.5% of the variance in overall satisfaction scores.

The unadjusted analysis showed notable variations in discharge-planning quality based on age, marital status, educational level, place of residence, and information source. Rural participants scored higher on discharge-planning quality than urban participants in the unadjusted analysis, with mean ratings of 7.8 and 6.0 and median scores of 8.0 and 7.0, respectively (*p* = 0.018). After adjustment, residency was not significantly associated with the quality of discharge planning. The urban residence coefficient (B = −1.342, β = −0.193, *p* = 0.081, 95% CI: −2.854 to 0.169) was negative and did not achieve statistical significance.

In the exploratory adjusted model, discharge-planning quality was associated with the source of information regarding endoscopy (B = 1.362, β = 0.303, *p* = 0.008, 95% CI: 0.367 to 2.357), but age, marital status, and educational level were not statistically significant. The attenuation of these connections implies that confounding or linkages between the predictors may have had an impact on the differences revealed in the unadjusted analysis. The statistically significant discharge-planning model explained 28.2% of the variance in the overall discharge-planning quality scores (R^2^ = 0.282, adjusted R^2^ = 0.229, F = 5.341, *p* < 0.001).‏ Because possible explanatory factors, including health literacy, access to follow-up care, and communication opportunities, were not specifically examined in this study, the relationship between urban residence and discharge-planning quality should be interpreted cautiously. Overall, in this sample, after adjustment, the quality of discharge planning was not associated with residence; however, the source of endoscopic information was statistically significantly associated with discharge-planning quality. Patient satisfaction was associated with prior endoscopic experience and endoscopic indication. These adjusted associations are exploratory findings and should not be interpreted as established independent determinants or predictors of either outcome; they require confirmation in larger, independent samples. These results imply that two related but different aspects of upper gastrointestinal endoscopy quality are patient satisfaction and discharge-planning quality.

## 5 Study limitations

This study has several important limitations to consider when interpreting the findings. First, the cross-sectional design and single-center setting limit the ability to establish causal relationships and reduce the generalizability of the results. Second, the relatively small sample size (*n* = 74) restricts the statistical power and the robustness of multivariable analyses. The multivariable analyses were secondary, exploratory, and hypothesis –generating and were not prospectively powered. Therefore, the adjusted estimates should not be interpreted as established independent determinants or predictors and require confirmation in larger, independent samples. Third, the use of a convenience sampling method may introduce selection bias and limit the representativeness of the study population. In addition, reliance on a self-administered electronic questionnaire may lead to response bias and exclude patients with limited digital literacy. Furthermore, patients reported their length of stay in the endoscopy unit, which may be subject to recall bias.‏ Because duration of stay was self-reported and objective arrival and discharge timestamps were unavailable, the reported median of 30 min should be interpreted as an approximate patient-reported estimate that may not fully capture the entire endoscopy-unit pathway. While the questionnaire was completed before participants left the endoscopy unit, judgments concerning the overall effectiveness of the discharge plan and the practical adequacy of activity instructions reflected immediate perceptions rather than verified post-discharge outcomes. Accordingly, these findings should not be interpreted as evidence of subsequent adherence, recovery, or effectiveness after returning home. Although an Aldrete score of 9–10 indicated physiological readiness for discharge, it did not establish complete cognitive recovery. Because sedation exposure was not systematically recorded, its potential influence on immediate self-reported comprehension could not be quantified or adjusted for. Therefore, the comprehension findings should be interpreted as patient-reported communication at the time of discharge rather than as evidence of sustained post-discharge recall or adherence. Finally, although the discharge planning tool demonstrated acceptable validity and reliability, it was newly developed and requires further validation in larger multicenter studies. Future prospective studies should evaluate and account for unmeasured variables such as sedation features, endoscopist experience, surgery type, biopsy status, post-procedure problems, and home accompaniment. We also note that the multivariable estimates may not have been as accurate and stable due to the small, single-center sample. As a result, we did not regard VIF values as proof of overall model stability and used them only to assess possible multicollinearity.

## 6 Conclusions

Patient satisfaction and the quality of discharge planning after upper gastrointestinal endoscopy were generally Favorable and positively associated in this single-center cross-sectional study. However, the observed association should not be interpreted as evidence that improved discharge planning or communication causes higher patient satisfaction because the cross-sectional design does not allow for causal inferences. In particular, the provision of written discharge instructions, dietary guidance, and post-discharge contact information was identified as a potential area for service improvement. Given the small convenience sample and the use of a recently developed tool for assessing discharge-planning quality, these results should be interpreted cautiously. The multivariable analyses were exploratory, secondary, and hypothesis-generating; they lacked prospective power. As a result, the adjusted connections need to be confirmed in larger, independent samples and should not be regarded as established independent determinants or predictors. Larger multicenter studies are needed to validate the tool and confirm these findings.

## 7 Implications for practice and research

The positive association between discharge-planning quality and patient satisfaction suggests that both should be considered in endoscopy quality-improvement efforts; however, the cross-sectional design does not establish causality. The relatively lower reporting of written instructions, dietary guidance, and post-discharge contact information identifies potential areas for service improvement. Waiting time should not be prioritized on the basis of these findings because it was well rated and was not associated with either outcome. The adjusted associations should not be taken as proven independent determinants or predictors because the multivariable analyses were secondary, exploratory, hypothesis-generating, and lacked predictive power. Larger multicenter longitudinal and intervention studies are needed to confirm these associations and to evaluate whether standardized discharge communication is associated with improvement in patient experience and post-discharge safety.

## Data Availability

The original contributions presented in the study are included in the article/Supplementary Material, further inquiries can be directed to the corresponding author.
