# Supplementary material for: Patient satisfaction and discharge-planning quality following upper gastrointestinal endoscopy: a single-center cross-sectional study
Source: Front Med (Lausanne). 2026 Oct 29;13:1903766. doi: 10.3389/fmed.2026.1903766 (PMC13754544; doi:10.3389/fmed.2026.1903766)
Supplement: Supplementary file 1 [file Datasheet1.docx]

**Supplementary Figure S1. Residual diagnostics for the patient-satisfaction regression model.**
