# Supplementary material for: Patient satisfaction and discharge-planning quality following upper gastrointestinal endoscopy: a single-center cross-sectional study
Source: Front Med (Lausanne). 2026 Oct 29;13:1903766. doi: 10.3389/fmed.2026.1903766 (PMC13754544; doi:10.3389/fmed.2026.1903766)
Supplement: Supplementary file 2 [file Datasheet2.docx]

|  |  |
| --- | --- |
|  | |

Supplementary Figure S2. Residual diagnostics for the discharge-planning-quality regression model.
