# Supplementary material for: Patient satisfaction and discharge-planning quality following upper gastrointestinal endoscopy: a single-center cross-sectional study
Source: Front Med (Lausanne). 2026 Oct 29;13:1903766. doi: 10.3389/fmed.2026.1903766 (PMC13754544; doi:10.3389/fmed.2026.1903766)
Supplement: Supplementary file 3 [file Supplementaryfile1.docx]

STROBE Statement—Checklist of Items for Cross-Sectional Studies

Patient Satisfaction and Discharge-Planning Quality Following Upper Gastrointestinal Endoscopy: A Single-Center Cross-Sectional Study

| Item | STROBE recommendation | Reported in manuscript / location | Status |
| --- | --- | --- | --- |
| 1a | Indicate the study’s design with a commonly used term in the title or abstract. | Title: “A Single-Center Cross-Sectional Study”; Abstract—Methods. | Yes |
| 1b | Provide an informative and balanced summary of what was done and what was found. | Abstract—Background, Aim, Methods, Results, Conclusions. | Yes |
| 2 | Explain the scientific background and rationale for the investigation. | Introduction. | Yes |
| 3 | State specific objectives, including any prespecified hypotheses. | Study aim and hypotheses; Objectives 1–3. | Yes |
| 4 | Present key elements of study design early in the paper. | Methods 2.1—Study Design and Setting. | Yes |
| 5 | Describe the setting, locations, and relevant dates, including recruitment, exposure, follow-up, and data collection periods. | Methods 2.1: Qassim University Medical City; 1 April–31 October 2024. | Yes |
| 6a | Give eligibility criteria, sources and methods of selection of participants, and methods of follow-up. | Methods 2.2—Participants and Eligibility; convenience sampling; inclusion/exclusion criteria; recruitment flowchart. | Yes |
| 6b | For matched studies, give matching criteria and number of exposed/unexposed. (If applicable.) | Not applicable—no matching was used. | N/A |
| 7 | Clearly define all outcomes, exposures, predictors, potential confounders, and effect modifiers; give diagnostic criteria, if applicable. | Methods 2.4—Instruments and study variables; satisfaction and discharge-planning measures; sociodemographic/clinical variables. | Yes |
| 8 | For each variable of interest, give sources of data and details of methods of assessment; describe comparability of assessment methods if more than one group. | Methods 2.4—Instruments; mGHAA-9 and study-specific discharge-planning questionnaire; data collection procedures. | Yes |
| 9 | Describe any efforts to address potential sources of bias. | Methods—standardized recruitment/data collection; anonymous electronic questionnaire. Biases also acknowledged in Limitations. | Yes |
| 10 | Explain how the study size was arrived at. | Methods 2.3—Sample Size: a priori calculation, estimated n=71.05 , rounded to 72; final n=74. | Yes |
| 11 | Explain how quantitative variables were handled in the analyses; if applicable, describe which groupings were chosen and why. | Methods—Statistical Analysis; descriptive summaries and non-parametric tests; regression analyses. | Yes |
| 12a | Describe all statistical methods, including those used to control for confounding. | Methods—Statistical Analysis: Mann–Whitney U, Kruskal–Wallis, Dunn post hoc, Spearman rho, multivariable linear regression. | Yes |
| 12b | Describe any methods used to examine subgroups and interactions. | Exploratory multivariable analyses reported; no interaction analysis was prespecified/reported. | Yes |
| 12c | Explain how missing data were addressed. | Methods—no missing responses were reported. | Yes |
| 12d | If applicable, describe analytical methods taking account of sampling strategy. | Convenience sampling is stated; no complex survey weighting was used. | N/A |
| 12e | Describe any sensitivity analyses. | sensitivity analyses were reported. | Yes |
| 13a | Report numbers of individuals at each stage of study—e.g., potentially eligible, examined for eligibility, confirmed eligible, included, and analysed. | Methods 2.2 and Figure 1: 85 underwent endoscopy; 80 eligible/approached; 6 declined; 74 analysed. | Yes |
| 13b | Give reasons for non-participation at each stage. | Methods 2.2: 5 excluded for age <18; 6 declined participation. | Yes |
| 13c | Consider use of a flow diagram. | Figure 1—Study participant recruitment flowchart. | Yes |
| 14a | Give characteristics of study participants and information on exposures and potential confounders. | Results—participant characteristics and descriptive tables. | Yes |
| 14b | Indicate number of participants with missing data for each variable of interest. | No missing responses reported. | Yes |
| 14c | Summarise follow-up time, if applicable. | Not applicable—cross-sectional design with no follow-up. | N/A |
| 15 | Report numbers of outcome events or summary measures over time, if applicable. | Results: median discharge-planning quality and satisfaction scores; percentages for discharge instruction indicators. | Yes |
| 16a | Give unadjusted estimates and, if applicable, confounder-adjusted estimates and precision (e.g., 95% CI). State which confounders were adjusted for and why. | Results—Spearman rho with 95% CI and p value; exploratory multivariable regression with covariates. | Yes |
| 16b | Report category boundaries when continuous variables were categorized. | Continuous measures were generally summarized/analyzed without categorization; categorical variables are defined in Results/Tables. | Yes |
| 16c | If relevant, consider translating estimates of relative risk into absolute risk for a meaningful time period. | Not applicable to the primary cross-sectional association. | N/A |
| 17 | Report other analyses done—e.g., analyses of subgroups and interactions, and sensitivity analyses. | Results—exploratory multivariable analyses and subgroup/comparative analyses as reported. | Yes |
| 18 | Summarise key results with reference to study objectives. | Results and Discussion/Conclusion. | Yes |
| 19 | Discuss limitations of the study, taking into account sources of potential bias or imprecision. | Discussion/Limitations: cross-sectional design, single center, small sample, convenience sampling, response/recall/digital-literacy issues, tool validation needs. | Yes |
| 20 | Give a cautious overall interpretation of results considering objectives, limitations, multiplicity of analyses, results from similar studies, and other relevant evidence. | Discussion and Conclusion; explicitly states association should not be interpreted as causation. | Yes |
| 21 | Discuss the generalisability (external validity) of the study results. | Limitations and Conclusion: findings limited by single-center convenience sample; need larger multicenter studies. | Yes |
| 22 | Give the source of funding and the role of the funders for the present study. | Funding and Acknowledgments: Deanship of Graduate Studies and Scientific Research, Qassim University (QU-APC-2026). | Yes |

Reference: von Elm E, Altman DG, Egger M, Pocock SJ, Gøtzsche PC, Vandenbroucke JP. The Strengthening the Reporting of Observational Studies in Epidemiology (STROBE) statement: guidelines for reporting observational studies. J Clin Epidemiol. 2008;61(4):344–349.
