# Supplementary material for: Patient satisfaction and discharge-planning quality following upper gastrointestinal endoscopy: a single-center cross-sectional study
Source: Front Med (Lausanne). 2026 Oct 29;13:1903766. doi: 10.3389/fmed.2026.1903766 (PMC13754544; doi:10.3389/fmed.2026.1903766)
Supplement: Supplementary file 4 [file Table2.docx]

**Supplementary Table S2. Descriptive statistics and primary analysis of the 8-item factual discharge-planning score and its association with patient satisfaction (N = 74).**

| **Measure** | **8-item factual discharge-planning score** |
| --- | --- |
| Minimum–maximum | 1.0–9.0 |
| Mean ± SD | 6.20 ± 2.18 |
| Median | 7.0 |
| IQR | 5.0–8.0 |
| Mean percentage score | 68.9 ± 24.2% |
| Median percentage score | 77.8% |
| IQR of percentage score | 55.6–88.9% |
| Sample size | 74 |

**Sensitivity analysis of the correlation with patient satisfaction**

| **Analysis** | **Spearman’s ρ** | **95% CI** | **p-value** | **N** |
| --- | --- | --- | --- | --- |
| Patient-satisfaction percentage score vs. 8-item factual discharge-planning percentage score | **0.633** | **0.468–0.756** | **<0.001** | **74** |
