# Supplementary material for: Patient satisfaction and discharge-planning quality following upper gastrointestinal endoscopy: a single-center cross-sectional study
Source: Front Med (Lausanne). 2026 Oct 29;13:1903766. doi: 10.3389/fmed.2026.1903766 (PMC13754544; doi:10.3389/fmed.2026.1903766)
Supplement: Supplementary file 5 [file Table3.docx]

**Supplementary Table S 3**. Sensitivity analyses using the 9-item and 10-item discharge-planning scores and their associations with patient satisfaction (N = 74).

| Measure | Original discharge-planning score (10 items) | Revised discharge-planning score (9 items) |
| --- | --- | --- |
| Minimum–maximum | 2.0–17.0 | 2.0–15.0 |
| Mean ± SD | 12.5 ± 3.9 | 11.1 ± 3.3 |
| Median | 14.0 | 12.0 |
| Interquartile range | 11.0–15.0 | 10.0–13.0 |
| Mean percentage score | 73.7% ± 23.1% | 73.9% ± 22.2% |
| Median percentage score | 82.4% | 80.0% |
| Interquartile range of percentage score | 64.7–88.2% | 66.7–86.7% |
| Sample size | 74 | 74 |

**Sensitivity analysis of the correlation with patient satisfaction**

| **Analysis** | **Spearman’s ρ** | **95% CI** | **p-value** | **N** |
| --- | --- | --- | --- | --- |
| Patient-satisfaction percentage score versus original 10-item discharge-planning percentage score | 0.630 | 0.463–0.753 | <0.001 | 74 |
| Patient-satisfaction percentage score versus revised 9-item discharge-planning percentage score | 0.595 | 0.418–0.728 | <0.001 | 74 |

**Notes:** The conditional comprehension item, “understanding of written instructions,” applied only to participants who received written instructions. For the sensitivity analysis, this item was excluded from the discharge-planning score for all participants, resulting in a revised 9-item score with a maximum possible score of 15. The original 10-item score had a maximum possible score of 17 when all items were applicable. Percentage scores were calculated using the relevant maximum possible score for each scoring approach. The positive association between patient satisfaction and discharge-planning quality remained statistically significant after exclusion of the conditional item, although Spearman’s ρ decreased slightly from 0.630 to 0.595. We estimated confidence intervals for Spearman’s ρ using Fisher’s r-to-z transformation, with the standard error calculated using the Fieller–Hartley–Pearson method.

**Abbreviations:** CI, confidence interval; IQR, interquartile range; SD, standard deviation; ρ, Spearman’s rank correlation coefficient.
