# Supplementary material for: Patient satisfaction and discharge-planning quality following upper gastrointestinal endoscopy: a single-center cross-sectional study
Source: Front Med (Lausanne). 2026 Oct 29;13:1903766. doi: 10.3389/fmed.2026.1903766 (PMC13754544; doi:10.3389/fmed.2026.1903766)
Supplement: Supplementary file 6 [file Table4.docx]

Supplementary Table S4. Residual and influence diagnostics for the multivariable linear regression model of patient satisfaction.

| **Residuals Statistics** | Minimum | Maximum | Mean | Std. Deviation | N |
| --- | --- | --- | --- | --- | --- |
| Predicted Value | 27.1335 | 36.6162 | 29.6216 | 2.93986 | 74 |
| Std. Predicted Value | -0.846 | 2.379 | 0.000 | 1.000 | 74 |
| Standard Error of Predicted Value | 0.924 | 2.274 | 1.337 | 0.483 | 74 |
| Adjusted Predicted Value | 26.9400 | 36.8366 | 29.6171 | 2.96608 | 74 |
| Residual | -12.13348 | 7.86652 | 0.00000 | 5.98226 | 74 |
| Std. Residual | -1.986 | 1.288 | 0.000 | 0.979 | 74 |
| Stud. Residual | -2.010 | 1.303 | 0.000 | 0.995 | 74 |
| Deleted Residual | -12.43198 | 8.06004 | 0.00448 | 6.18360 | 74 |
| Stud. Deleted Residual | -2.056 | 1.310 | -0.002 | 1.001 | 74 |
| Mahal. Distance | 0.682 | 9.130 | 2.959 | 2.912 | 74 |
| Cook's Distance | 0.000 | 0.065 | 0.008 | 0.010 | 74 |
| Centered Leverage Value | 0.009 | 0.125 | 0.041 | 0.040 | 74 |
