# Supplementary material for: Patient satisfaction and discharge-planning quality following upper gastrointestinal endoscopy: a single-center cross-sectional study
Source: Front Med (Lausanne). 2026 Oct 29;13:1903766. doi: 10.3389/fmed.2026.1903766 (PMC13754544; doi:10.3389/fmed.2026.1903766)
Supplement: Supplementary file 7 [file Table5.docx]

Supplementary Table S5. Residual and influence diagnostics for the multivariable linear regression model of discharge-planning quality.

| Residuals Statistics | Minimum | Maximum | Mean | Std. Deviation | N |
| --- | --- | --- | --- | --- | --- |
| Predicted Value | 4.3502 | 8.7318 | 6.2027 | 1.15598 | 74 |
| Std. Predicted Value | -1.603 | 2.188 | 0.000 | 1.000 | 74 |
| Standard Error of Predicted Value | 0.363 | 1.130 | 0.514 | 0.182 | 74 |
| Adjusted Predicted Value | 2.7899 | 8.9182 | 6.1883 | 1.22447 | 74 |
| Residual | -3.75643 | 3.64977 | 0.00000 | 1.84469 | 74 |
| Std. Residual | -1.965 | 1.910 | 0.000 | 0.965 | 74 |
| Stud. Residual | -2.014 | 2.282 | 0.003 | 1.007 | 74 |
| Deleted Residual | -3.94419 | 5.21006 | 0.01437 | 2.01616 | 74 |
| Stud. Deleted Residual | -2.061 | 2.357 | 0.002 | 1.019 | 74 |
| Mahalanobis Distance | 1.653 | 24.546 | 4.932 | 4.906 | 74 |
| Cook's Distance | 0.000 | 0.371 | 0.016 | 0.044 | 74 |
| Centered Leverage Value | 0.023 | 0.336 | 0.068 | 0.067 | 74 |
